# Flexibility of temporal expectations for triple subdivision of a beat

**DOI:** 10.2478/v10053-008-0063-7

**Published:** 2009-04-27

**Authors:** Bruno H. Repp, Haitham Jendoubi

**Affiliations:** 1 Haskins Laboratories, New Haven, Connecticut; 2 Cognitive Science Program, Yale University, New Haven, Connecticut

**Keywords:** synchronization, subdivision, timing, expectation, phase correction

## Abstract

When tapping in synchrony with an isochronous sequence of beats, participants respond automatically to an unexpectedly early or late beat by shifting their next tap; this is termed the phase correction response (PCR). A PCR has also been observed in response to unexpected perturbations of metrical subdivisions of a beat, which suggests that participants have temporal expectancies for subdivisions to occur at particular time points. It has been demonstrated that a latent temporal expectancy at 1/2 of the inter-beat interval (IBI) exists even in the absence of explicit duple subdivision in previous IBIs of a sequence. The present study asked whether latent expectancies at 1/3 and 2/3 of the IBI can be induced by a global experimental context of triple subdivision, and whether a local context of consistently phase-shifted triple subdivisions can induce different expectancies. Using the PCR as the dependent variable, we find weak evidence for latent expectancies but strong evidence for context-induced shifts in expectancies. These results suggest that temporal referents between beats, which typically are linked to simple ratios of time spans, are flexible and context-dependent. In addition, we show that the PCR, a response to expectancy violation, is independent of and sometimes contrary to the simultaneous phase adaptation required by a change in subdivision timing.

## Introduction

Entrainment of movement to a periodic acoustic stimulus has been the subject of many studies attempting to specify the relationship between auditory perception and rhythmic action. Some research has been devoted to developing models that predict the phase of tapping as a function of the phase of the previous beat(s) in the sequence (Mates, 1994a, 1994b; Pressing, 1998; Vorberg & Schulze, 2002). Other related studies describe attention or movement as being driven by internal oscillators that are entrained by the stimulus sequence (Jones & Boltz, 1989; Large, 2000; Large & Jones, 1999; Large & Kolen, 1994). A third, less model-oriented line of research introduces timing perturbations in a sequence and examines participants’ responses to them (Repp, 2001, 2002a, 2008a; see Repp, 2005, for a review). For example, a sequence of beats to which a participant is tapping synchronously is phase-shifted at some point and the phase shift of the tap following the first shifted beat is measured. This measure is called the *phase correction response* (PCR) and constitutes a simple index of sensorimotor coupling.

**Figure 1.**
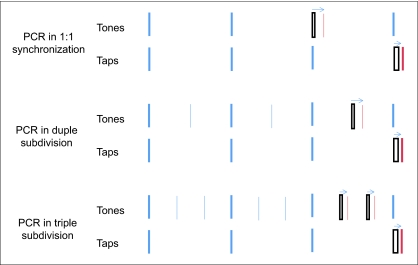
Schematic illustration of the phase correction response (PCR) in simple and subdivided sequences. Thick vertical bars represent beat tones and taps, thin vertical bars represent subdivision tones. Outline bars represent a tone that has been perturbed or a tap that has undergone a phase shift, in the direction of the arrow above the sequence.

Repp (2008a) recently demonstrated that a PCR is elicited not only by a phase-shifted beat but also by phase-shifted subdivisions of an unperturbed beat. Figure 1 illustrates schematically three of the conditions in his study. On top is the standard situation: One tone in a series of simple beat tones is shifted (delayed, in this example), and the next tap is observed to shift automatically in the same direction, though typically by less than the shift of the tone. The second display shows a sequence of beats with duple subdivision, where subdivision tones occur at 1/2 of the inter-beat interval (IBI). If one of the subdivision tones is shifted, this elicits a PCR in the next tap, even though the taps are synchronized with the beats, not the subdivisions. The third display shows a sequence with triple subdivision of the beat, where the subdivision tones occur at 1/3 and 2/3 of the IBI. A simultaneous shift of the two subdivision tones again elicits a PCR. These effects suggest that participants are perceptually monitoring the subdivision tones as well as the beats and are using all of them as temporal references for placing each tap. (See also Large, Fink, & Kelso, 2002, for a similar argument.)

Repp’s (2008a) study included a number of additional conditions, one of which (“local subdivision”) is of particular interest here. In that condition, a single subdivision tone appeared unexpectedly in a sequence of simple (i.e., not subdivided) beats. If that tone occurred at 1/2 of the IBI, the next tap shifted very little, but if the tone occurred slightly earlier or later, a PCR was elicited. This finding suggested that participants had a *latent expectation* of duple subdivision: It seemed as if they compared the time of occurrence of the subdivision tone to the expected time point (1/2 of the IBI) and reacted to any discrepancy with a PCR. Indeed, connectionist and coupled-oscillator models of rhythm perception (Desain, 1992; Large, 2000) predict that harmonics (1/2, 1/3, 1/4) of a beat period will be entrained together with the beat period, albeit more weakly, with latent expectations being the consequence.

In the present study we started by asking three questions. First, can participants have latent expectancies of triple subdivision? Music theoretic descriptions of rhythm generally assume a propensity of listeners to mentally divide time spans into two equal parts (Drake & Bertrand, 2001), and there is evidence that infants, children, and adults have more difficulty with triple than with duple meter (Bergeson & Trehub, 2006; Drake, 1997; Repp, 2003a). However, this does not preclude a weaker propensity to divide time spans into thirds. One potential problem, though, is that latent expectations of duple and triple subdivision are mutually exclusive. If latent expectations of duple subdivision are the default mode, evidence for latent expectations of triple subdivision might be difficult to obtain unless participants are given a good reason for having such expectations. We encouraged these expectations by embedding our test sequences in a global experimental context that exposed participants to various forms of triple subdivision.

Our second question was whether expectancies of triple subdivision are strongly linked to the 1/3 and 2/3 points or whether they can be adapted rapidly to a local context of phase-shifted subdivisions. Oscillator models such as dynamic attending theory (Large & Jones, 1999) and models that presuppose simple-ratio cognitive biases such as quantization (Desain, 1992) predict a strong preference for subdivisions that divide a beat into intervals that form a simple integer ratio. If so, then if subdivisions were shifted consistently from their standard metrical positions, expectations might not shift with them or might shift only very gradually. Alternatively, phase-shifted subdivisions might quickly be expected to occur in their new, shifted positions. Although deviations from simple-ratio timing are common in musical practice (for example, in the “swing rhythm” of jazz performance; see Friberg & Sundström, 2002; Honing & de Haas, 2008), it could be argued that the aesthetic effect of such timings derives from the fact that they are perceived as deviations from simple-ratio expectations. If that were the case, subdivisions occurring unexpectedly on time (i.e., at the 1/3 and 2/3 points) in a local context of phase-shifted subdivisions should not elicit a PCR. However, if expectations adapt quickly to local context, then on-time subdivisions should elicit a PCR. We tested this prediction in our experiments.

Third, in order to examine the relative salience of the first (1/3) and second (2/3) triple subdivision points, and to see whether a single “triple subdivision” tone is sufficient to induce temporal expectancies and elicit a PCR when shifted, we manipulated the configuration of subdivisions: first subdivision only (S1), second subdivision only (S2), or both (S12). In the triple subdivision condition of Repp’s (2008a) study, both subdivisions were always present, but they were shifted either singly or jointly. Shifting only S1 did not elicit a PCR, probably because the following S2 neutralized it. Shifting S2 elicited a PCR only at the slower of two tempi used (IBI = 540 or 720 ms), whereas shifting S12 elicited a PCR at both tempi. We used an IBI of 720 ms here to avoid possible rate limits on the sensorimotor effects of subdivisions (Repp, 2003a) and examined the effects of shifting either subdivision tone in the absence of the other, as well as shifting both together.

In Experiment 1 we used an event-onset-shift paradigm (Repp, 2002a, 2005): In short sequences of beat tones, one or two subdivision tones either occurred just once (to test latent expectations) or started with a particular timing (local context), then shifted relative to the context, and then immediately shifted back to the context timing. This design focused on the PCR to the critical subdivision tone(s). However, it became clear in the course of the experiment that in order to be able to interpret the PCR as an index of temporal expectations, it is necessary to demonstrate its independence of any changes in asynchronies (tapping phase) that are caused by a change in timing of subdivisions. (We will explain this issue in more detail below.) To gain a more comprehensive view of these changes, we subsequently conducted Experiment 2, in which we used a phase-shift paradigm and longer sequences.

## EXPERIMENT 1

### Methods

#### Participants

The participants included 8 graduate students from the Yale School of Music (5 women, 3 men, ages 22-28), who were paid for their services, and the two authors (ages 63 and 21, respectively). All participants had substantial music training and (except for author H.J.) were regular participants in synchronization experiments.

#### Materials and equipment

Each sequence (trial) consisted of a series of 11 beat tones with a constant IBI of 720 ms. The first two IBIs were always empty; the following five IBIs were context IBIs that were either empty or contained subdivision tones that were on-time, early, or late relative to the 1/3 and 2/3 points of the IBI; and the subsequent IBI was the probe IBI that likewise contained on-time, early, or late subdivisions. The probe IBI was followed by two context IBIs identical to the five preceding it. On-time subdivisions occurred at 240 ms and/or 480 ms after the beat. Early subdivisions occurred 60 ms earlier, at 180 ms and/or 420 ms after the beat. Late subdivisions occurred 60 ms later, at 300 ms and/or 540 ms after the beat. The factorial combination of three subdivision types (S1, S2, or S12), four context conditions (early, on-time, late, or none), and three probe timings (early, on-time, or late) resulted in 36 different sequences that were presented eight times in different random orders (generated anew for each participant).

A program written in MAX 4.0.9, running on an Intel iMac computer, controlled the experiment. The tones (piano timbre) were produced by a Roland RD-250s digital piano according to musical-instrument-digital-interface (MIDI) instructions from the MAX program. Beat tones were sounded at B-flat7 (3729 Hz) and subdivision tones one semitone lower, at A7 (3520 Hz). This pitch difference was sufficient to distinguish the tones and was kept small to avoid auditory stream segregation (Bregman, 1990). All tones had nominal durations of 40 ms. Audio output was presented over Sennheiser HD540 II headphones. Participants tapped with the index or middle finger of their preferred hand on a Roland SPD-6 percussion pad that was held on the lap.

#### Procedure

Participants sat in front of a computer monitor that showed instructions and the number of trials elapsed in the block. After receiving instructions, they started each trial by pressing the space bar of the computer keyboard, commenced tapping with the third beat tone, and continued to tap in synchrony with the beats while ignoring the subdivisions. Participants had the (rarely used) option of repeating a trial by clicking a button on the screen. There were short breaks between blocks during which the data were saved. The experiment lasted about one hour.

#### Analysis

Asynchronies were computed by subtracting the times of occurrence of beat tones from those of the coincident taps. An additional 15 ms was subtracted to take previously measured electronic processing delays into account. Some asynchronies that were obvious outliers (probably due to inattention) were deleted. Occasionally, taps were missing due to insufficient tapping force. The total percentage of trials affected by such problems was less than 0.5. The PCR in each trial was calculated by subtracting the pre-probe asynchrony (the asynchrony of the tap immediately preceding the probe) from the post-probe asynchrony (the asynchrony of the tap immediately following the probe). This is equivalent to subtracting the IBI from the interval between the pre- and post-probe taps. Asynchrony and PCR data were averaged over the eight repetitions of each trial type. The data were submitted to repeated-measures ANOVAs, separating the no-context condition from the other context conditions. The Greenhouse-Geisser correction was applied to all p values.

### Results and discussion

#### Phase correction responses

##### No-context condition

If participants have latent expectations for triple subdivision in the no-context condition, an early probe should elicit a negative PCR (tap advancement), a late probe a positive PCR (tap delay), and an on-time probe no PCR, regardless of type of subdivision (S1, S2, or S12). If participants have no latent expectations, none of the probes should elicit a PCR. A third possibility is that, despite the global experimental context of triple subdivision, participants revert to a default latent expectation of duple subdivision (at 360 ms after the beat) in the no-context condition. In that case, all S1 probes (occurring at 180, 240, or 300 ms) should elicit negative PCRs, all S2 probes (occurring at 420, 480, or 540 ms) should elicit positive PCRs, and S12 probes should elicit hardly any PCR. It is also possible that early S1 probes (at 180 ms) and late S2 probes (at 540 ms) would not elicit any PCR if duple subdivision is expected because they coincide with points of quadruple subdivision (1/4 and 3/4 of the IBI).

**Figure 2.**
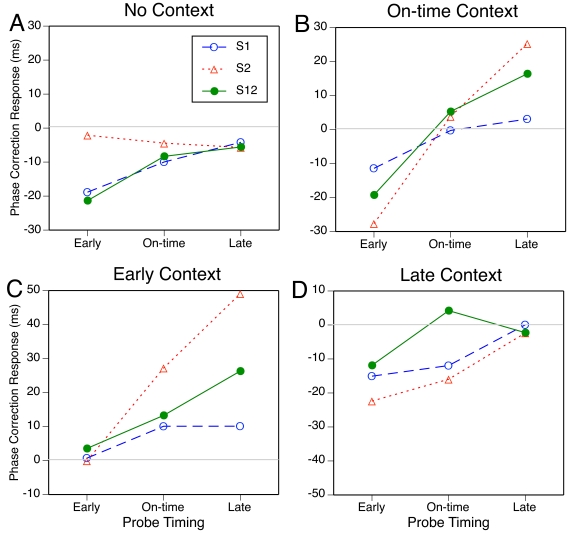
The mean phase correction response to probes in the four context conditions, as a function of subdivision type and probe timing.

The results are shown in Figure 2 (A). They do not correspond to any of the three scenarios outlined above. All PCRs were negative, reflecting a forward shift of the critical tap. S1 and S12 elicited increasingly negative PCRs as they were shifted forward in time, but S2 did not. In the ANOVA, the main effect of subdivision type, *F*(2, 18) = 4.1, *p* = .039, and the interaction with probe timing, *F*(4, 36) = 3.2, *p* = .035, reached significance; the main effect of probe timing did not.

The PCR results for S2, which are rather close to zero, suggest that participants did not have any temporal expectations for S2. Consistent with this interpretation, the similarity of the PCR functions for S1 and S12 suggests that the effect of S12 was due to S1 alone, with no contribution from S2. Participants did seem to have a latent expectation for S1 because probe timing had an effect with S1 and S12. However, the negativity of the PCRs for on-time and late S1 and S12 probes poses a problem for interpretation. If participants’ expectations had been centered on the 1/3 point, the PCR to late S1 probes should have been positive and that to on-time S1 probes should have been near zero. If expectations had been centered instead on the 1/2 point, which would be compatible with the S1 and S12 results, S2 probes should have elicited positive PCRs.

One reasonable possibility is that the appearance of any local subdivision, regardless of its timing, elicited a small negative shift of the next tap. This could be regarded as a constant error, a kind of surprise reaction. Repp (2008a) likewise found a small negative shift in response to a local on-time duple subdivision, although this detail was not mentioned in the published article. If all the data points in Figure 2 (A) were imagined as shifted upward by about 10 ms, so that on-time S1 and S12 probes have a zero effect, the results would be compatible with a latent expectation centered on the 1/3 point. The slightly positive PCR in response to early S2 probes then could be regarded as a result of these probes being perceived as very late with respect to the 1/3 point, and on-time and late S2 probes as being too distant from that single reference point to elicit any PCR. An alternative possibility is that latent expectations for S1 were not centered on the 1/3 point but on a point about 10 ms earlier. This would imply that participants’ expectations deviated from simple interval ratios.

##### On-time context condition

In the on-time context condition, participants were expected to have strong expectations of on-time subdivisions, so that the probe, regardless of type, would elicit a negative PCR when early, a positive PCR when late, and no PCR when on time. These expectations were confirmed by the results, shown in Figure 2 (B). However, there was a clear difference among subdivision types: S2 probes elicited the strongest PCRs, S1 probes the weakest, and S12 probes fell in between. ANOVA showed these differences to be highly reliable: Both the main effect of probe timing, *F*(2, 18) = 30.4, *p* < .001, and the interaction with subdivision type, *F*(4, 36) = 11.8, *p* < .001, were significant.

So, in contrast to the no-context condition, participants seemed to have stronger expectations for S2 than for S1 in the on-time context. Alternatively, they may have reacted more strongly to a shifted S2 than to a shifted S1 because S2 was perceptually grouped with the following beat tone, the synchronization target. There was also an asymmetry in the response to early versus late probes, with PCRs to early probes being larger. (Note a similar tendency in the no-context condition, Figure 2 [A].)

##### Early context condition

In this condition, early probes were not expected to elicit a PCR because they merely continued the context. Late probes were expected to elicit positive PCRs because they were late both relative to the context and relative to any lingering expectations of on-time subdivisions. The responses to on-time probes were of primary interest: On-time probes should elicit a positive PCR if expectations adapt to the local context, but no PCR if expectations do not adapt. They did elicit a positive PCR, as Figure 2 (C) shows. Again, however, participants responded much more strongly to S2 probes than to S1 probes, with S12 probes falling in between. In the ANOVA, the main effects of subdivision type, *F*(2, 18) = 8.3, *p* = .003, and probe timing, *F*(2, 18) = 28.8, *p* < .001, as well as the interaction, *F*(4, 36) = 9.8, *p* < .001, were significant. These results suggest that participants had formed expectations of early subdivisions, against which the probes were compared.

##### Late context condition

In this condition, late probes were not expected to elicit a PCR because they merely continued the context. Early probes were expected to elicit negative PCRs. On-time probes should elicit negative PCRs if expectations adapt to local context, but no PCRs if expectations do not adapt. The results, shown in Figure 2 (D), show that on-time S1 and S2 probes elicited negative PCRs, but an on-time S12 probe did not. Also, the tendency to respond more strongly to S2 probes than to S1 probes was much smaller here than in the on-time and early context conditions. ANOVA revealed significant main effects of subdivision type, *F*(2, 18) = 13.4, *p* < .001, and probe timing, *F*(2, 18) = 16.4, *p* < .001, but no significant interaction, *F*(4, 36) = 2.5, *p* = .085. The results are consistent with the formation of context-induced expectations for late subdivisions if they occur singly. Late S12 context, however, did not seem to induce expectations of late subdivisions, for whatever reason. Later, in Experiment 2, we will argue that this conclusion is probably too strong.

##### Comparing on-time, early, and late context conditions

In an overall three-way ANOVA on the on-time, early, and late context conditions, all main effects and interactions were significant, which confirms the reliability of the differences in response pattern for different context conditions. We also compared the results across context conditions separately for each subdivision type. In each of these three ANOVAs, the main effects of context condition and probe timing obviously were significant. In addition, however, the interaction was also significant for S2, *F*(4, 36) = 6.9, *p* = .002, and for S12, *F*(4, 36) = 5.7, *p* = .007, though not for S1, *F*(4, 36) = 1.3, *p* = .291. It can be seen in Figure 2 (B, C, and D) that for both S2 and S12 the PCR function was much less steep in the late context condition than in the on-time and early context conditions, whereas for S1 there was little difference. Thus it seems that early and on-time contexts induced stronger expectations for S2 than did a late context, whereas expectations for S1 were relatively weak in all contexts, if indeed the PCRs reflect the violation of temporal expectations.

#### Pre-probe asynchronies

**Figure 3.**
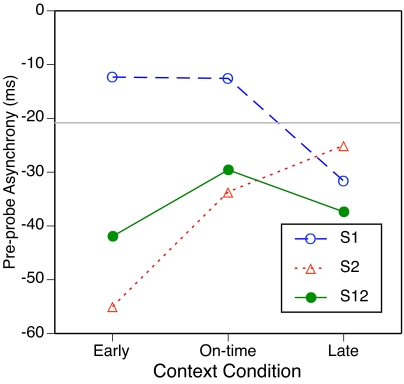
T he mean pre-probe asynchrony as a function of subdivision type and context condition. The grey horizontal line represents the mean pre-probe asynchrony in the no-context condition.

The reason why it is not wise to jump to conclusions regarding participants’ expectations in the shifted-context conditions is that the PCR represents the difference between the (immediate) post-probe and pre-probe asynchronies and thus depends on the magnitude of the pre-probe asynchrony. If phase-shifted context affected the pre-probe asynchrony, the PCR may not (or not only) reflect an effect of temporal expectancy violation by the probe but rather (or also) an incipient change from a context-specific asynchrony to a probe-specific asynchrony. We will refer to this change as *phase adaptation*. Phase adaptation may be independent of any cognitive temporal expectations that participants may have. If phase adaptation fully accounted for the PCR, no conclusions could be drawn about participants’ expectations, which could well have remained unaffected by context, even though this seems highly unlikely. Therefore, we examined the pre-probe asynchrony as a function of context condition and subdivision type, averaging over the three probe timings. (Probe timing naturally could not have any effect on the pre-probe asynchrony; this was confirmed in the ANOVAs, where probe timing was included as a variable but was not involved in any significant effects.) Figure 3 shows the results.

As is commonly found, all mean asynchronies were negative, meaning that the pre-probe tap generally preceded the pre-probe beat tone. The grey horizontal line represents the mean pre-probe asynchrony in the no-context condition (-21 ms). Relative to this baseline, on-time or early S1 context moved the pre-probe tap a bit closer to the pre-probe beat tone, whereas on-time S2 or S12 context increased the lead of the tap. These effects could be understood as an attraction of the tap to the nearest subdivision tone, although earlier studies using target-distractor paradigms (Repp, 2003b, 2004) have suggested that such attraction occurs only within time windows of ±150 ms. Early S2 context advanced the tap even more, whereas late S2 context shifted it little. By contrast, early S1 context delayed the tap by as much as did on-time S1 context, but late S1 context actually advanced the tap. S12 context generally advanced the tap, without much difference between early and late conditions.

This curious pattern of effects of shifted subdivisions on tapping phase was quite reliable. In the ANOVA, the main effects of subdivision type, *F*(2, 18) = 20.0, *p* < .001, and context condition, *F*(2, 18) = 5.6, *p* = .013, as well as the interaction, *F*(4, 36) = 16.6, *p* < .001, were significant. Separate ANOVAs on each subdivision type confirmed significant effects of context condition for S1, *F*(2, 18) = 8.5, *p* = .003, and S2, *F*(2, 18) = 41.3, *p* < .001, but not for S12, *F*(2, 18) = 2.6, *p* = .104.

#### Prediction of PCRs

The pattern of pre-probe asynchronies can be used to predict the pattern of PCRs on the assumption that each PCR represents the incipient change from a mean asynchrony associated with the context pattern to a mean asynchrony associated with the probe pattern. The latter can be estimated by the mean pre-probe asynchrony for the context pattern that is identical with the probe pattern. Thus, for example, the fact that early and on-time S1 contexts led to almost identical mean pre-probe asynchronies (Figure 3)predicts a zero PCR when an early S1 probe occurs in an on-time S1 context, or the reverse. However, these conditions actually yielded small negative PCRs, as can be seen in Figure 2 (B and C). The fact that a late S1 context led to a more negative pre-probe asynchrony than did an early or on-time S1 context (Figure 3) implies that a late S1 probe in an early or on-time S1 context should elicit a *negative* PCR, whereas an early or on-time probe in a late S1 context should elicit a *positive* PCR. Both predictions are counterintuitive and are not confirmed by the data in Figure 2. The predictions for S12 also run into difficulties: The similar pre-probe asynchronies for early and late S12 contexts (Figure 3) suggest that no PCR should be obtained for early S12 probes in late S12 contexts and vice versa, but this is not what the data in Figure 2 show. Thus it seems that the PCR cannot be explained simply as an incipient change from one context-specific mean asynchrony to another. The violation of perceptual expectancies by the probe seems to have had an independent effect on the PCR. However, phase adaptation may have played a role, too.

To determine the relative contributions of these two predictor variables (expectancy violation and phase adaptation) we conducted a stepwise multiple regression analysis on the three context conditions combined (27 data points). The dependent variable was the mean PCR for each condition. The predictor variable for phase adaptation was obtained by subtracting the mean pre-probe asynchrony for a given context from the mean pre-probe asynchrony for the context that corresponded to a given probe (as described in the preceding paragraph). The predictor variable for expectancy violation was the magnitude of the temporal shift between context and probe (ranging from -120 to 120 ms). Although both predictor variables were positively correlated with the PCR, expectancy violation was the stronger predictor, accounting for 72.7% of the variance, *t*(26) = 10.21, *p* < .001. However, phase adaptation accounted for a significant additional 14.7% of the variance, *t*(26) = 5.17, *p* < .001, about half of the residual variance. Together the two predictors thus accounted for a healthy 87.1% of the variance in the mean PCRs. Because the constant in the regression equation (2.1 ms) was not significantly different from zero, as should be the case, the regression coefficients (.19 and .49, respectively) can be interpreted as proportions. Thus it can be concluded that the PCR reflects about 20% of the expectancy violation plus about 50% of the (generally much smaller) phase adaptation.

It is also quite clear that the PCRs in the no-context condition (Figure 2 [A]) do not represent changes from the mean no-context pre-probe asynchrony (the grey horizontal line in Figure 3) to the various context-specific asynchronies (data points in Figure 3), regardless of whether or not an overall negative shift in response to no-context probes is taken into account. For example, an early S2 probe in the no-context condition should have elicited a clear negative PCR (Figure 3), but it did not (Figure 2 [A]). The data patterns in Figure 2 (A) and 3 are contradictory, and only an explanation in terms of latent expectancies for S1 seems feasible for the no-context PCR data.

## EXPERIMENT 2

The design of Experiment 1, employing short sequences and timing perturbations of the event-onset-shift type, focused on the PCR but did not permit a close examination of phase adaptation (the trajectory of asynchronies) between two subdivision regimes. Because the shifted subdivisions immediately shifted back to their context configuration, the adaptation (or its beginning) coincided with the PCR elicited by the expectancy violation. Furthermore, given that the pre-probe context was repeated only five times, it is possible that participants had not yet adapted completely to the context by the time the probe occurred. Finally, it is conceivable that in some conditions (such as a late S2 probe) the PCR was actually delayed by one tap due to the short interval between the probe and the post-probe tap. Such a delay was difficult to detect given that the timing of the post-probe subdivisions reverted to that of the pre-probe context and thus may have caused a second PCR that would have tended to cancel a delayed PCR.

To address these concerns, Experiment 2 employed longer sequences and a phase-shift paradigm in which one temporal pattern of subdivisions (or empty IBIs) shifted to another pattern (or empty IBIs) in the middle of the sequence. This gave us the opportunity to observe the full phase adaptation as well as the PCR elicited by expectancy violation at the point of change. Because the first point of change (the *probe*) is identical in event-onset-shift and phase-shift paradigms, the PCR and pre-probe asynchrony results of Experiment 2 were expected to replicate those of Experiment 1. However, several new questions could be asked in Experiment 2. One question was whether there would be any instances of delayed PCR. A second question was whether there are any long-term effects of the initial subdivision pattern on the asynchronies with the final subdivision pattern. In other words, how many taps does it take before the asynchronies with a final pattern reach an asymptote that is independent of the preceding initial pattern? Third, the time course of phase adaptation to the initial pattern could be examined as well, to confirm that adaptation is complete by the time the phase shift occurs. Finally, Experiment 2 included a new condition, involving changes from subdivisions to empty IBIs (a no-probe condition, as it were). Would the sudden cessation of subdivisions elicit a PCR?

Because the terms *context* and *probe* seem less appropriate to the new design, we adopt a new terminology: The initial configuration of subdivisions or empty IBIs (previously called the context) is called *pattern A*, and the subsequent configuration is called *pattern B*. The IBI in which *pattern B* starts (previously called the *probe*) is called the *A-B transition*.

### Methods

#### Participants

The participants included 9 graduate students from the Yale School of Music (6 women, 3 men, ages 22-28), who were paid for their services, and author B.H.R. All were regular participants in synchronization experiments. Three of the musicians and B.H.R. had participated in Experiment 1, about 9 months earlier.

#### Materials and equipment

Each sequence (trial) consisted of a series of 22 beat tones with a constant IBI of 720 ms. The first two IBIs were always empty; the following nine IBIs were either empty or contained on-time, early, or late subdivisions (pattern A); and the remaining ten IBIs likewise were either empty or contained on-time, early, or late subdivisions (pattern B). The factorial combination of three subdivision types (S1, S2, or S12), four A-patterns, and four B-patterns resulted in 46 different sequences that were presented four times in different random orders (generated anew for each participant). Timing, pitch, and relative intensity of the tones, as well as the equipment used, were the same as in Experiment 1.

#### Procedure

The procedure was the same as in Experiment 1.

#### Analysis

The analysis was also similar to that in Experiment 1, except that mean asynchronies were computed for all taps. The conditions containing empty IBIs as either the A- or the B-pattern were treated separately from the other conditions. The condition in which both patterns consisted of empty IBIs was excluded from most analyses as it provided little information. (That condition occurred three times in the factorial design but was presented only once; hence the total number of 46 different sequences.)

### Results and discussion

#### Phase correction responses

**Figure 4.**
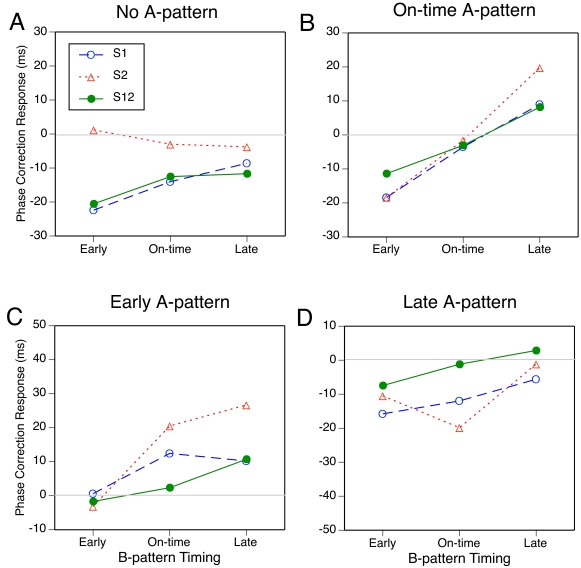
The mean phase correction response to the A-B transition for four A-patterns as a function of subdivision type and B-pattern timing.

To facilitate comparisons with Experiment 1, the mean PCRs for the shared conditions are shown in Figure 4, which has the same format as Figure 2. Overall, PCRs were somewhat smaller than in Experiment 1, especially when pattern A was on time or early, but the pattern of results resembles that in Figure 2. 

The resemblance is especially close in the conditions with an empty A-pattern (Figure 4 [A]). As in Experiment 1, all PCRs for S1 and S12 were negative and depended on B-pattern timing (the earlier the subdivisions occurred, the more negative was the PCR), whereas PCRs for S2 were barely different from zero and unaffected by B-pattern timing. The main effect of subdivision type was significant, *F*(2, 18) = 22.3, *p* < .001, as was the interaction with B-pattern timing, *F*(4, 36) = 4.0, *p* = .024, just as in Experiment 1. A joint ANOVA of both experiments (treating the two participant groups as independent) yielded in addition a significant main effect of B-pattern (probe) timing, *F*(2, 36) = 5.4, *p* = .009, but no significant effect involving experiment.

When pattern A was on time (Figure 4 [B]), PCRs were negative for early B and positive for late B, as expected. The differences between subdivision types were less clear here than in Experiment 1, however. Compared to Experiment 1, participants in Experiment 2 responded more vigorously to a shifted S1 and less vigorously to a shifted S2 or S12. In the ANOVA, only the main effect of B-pattern timing was significant, *F*(2, 18) = 36.6, *p* < .001. In a joint ANOVA of both experiments, the interaction with subdivision type was significant as well, *F*(4, 72) = 6.9, *p* < .001, and the triple interaction with experiment reached significance, *F*(4, 72) = 3.0, *p* = .032, because the two-way interaction was more pronounced in Experiment 1 than in Experiment 2.

With the early A-pattern (Figure 4 [C]), too, PCRs to shifts of S2 or S12 were weaker here than in Experiment 1, whereas PCRs to a shifted S1 were of comparable size. All PCRs to on-time and late B-patterns were positive, as expected. In the ANOVA, the main effect of B-pattern timing was most pronounced, *F*(2, 18) = 15.2, *p* = .001, but the main effect of subdivision type, *F*(2, 18) = 5.1, *p* = .035, and the interaction, *F*(4, 36) = 3.3, *p* = .050, reached significance as well. In a joint ANOVA of the two experiments, all three effects were highly reliable, but there was no significant interaction involving experiment. The main effect of experiment reached significance, *F*(1, 18) = 6.0, *p* = .025, due to generally smaller PCRs in Experiment 2.

When the A-pattern was late (Figure 1 [D]), PCRs to early and on-time B-patterns were negative, as expected. Surprisingly, a shift from a late to an early S2 elicited a less negative PCR than did a shift to an on-time S2. In the ANOVA, only the interaction was significant, *F*(4, 36) = 10.9, *p* = .001. In a joint ANOVA of the two experiments, however, the main effects of subdivision type, F (2, 36) = 6.8, *p* = .006, and of B-pattern (probe) timing, *F*(2, 36) = 5.6, *p* = .013, were significant as well, as were the interactions of experiment with subdivision type, *F*(2, 36) = 5.6, *p* = .011, with B-pattern (probe) timing, *F*(2, 36) = 12.6, *p* < .001, and with both of these variables, *F*(4, 72) = 6.6, *p* = .001. In this case then, the pattern of results was really different in the two experiments, though the reasons for this are unclear.

In an overall 3 x 3 x 3 ANOVA on the data of Figure 4 (panels B-D), the main effects of A-pattern timing, *F*(2, 18) = 35.3, *p* < .001, and of B-pattern timing, *F*(2, 18) = 40.5, *p* < .001, were highly significant, and the interaction was significant as well, *F*(4, 36) = 7.0, *p* = .002. The interaction seemed to be due in large part to reduced PCRs when A- and B-patterns were 120 ms apart, compared to when the shift was only 60 ms. This may reflect a nonlinearity in the PCR as a function of the magnitude of the expectancy violation (cf. Repp, 2002b). Of the other effects, only the interaction of subdivision type and A-pattern timing reached significance, *F*(4, 36) = 7.3, *p* < .001: Effects of A-pattern timing were larger for S2 than for S1 and S12. Separate ANOVAs on each subdivision type showed significant main effects of A-pattern timing for S1, *F*(2, 18) = 22.1, *p* < .001, and S2, *F*(2, 18) = 43.0, p < .001, but not for S12; significant main effects of B-pattern timing for S1, *F*(2, 18) = 10.7, *p* = .002, S2, *F*(2, 18) = 43.4, *p* < .001, and S12, *F*(2, 18) = 9.5, *p* = .005; and a significant interaction only for S2, *F*(4, 36) = 8.1, *p* = .001, for which the reduction in the PCR to large phase shifts was most pronounced. Joint ANOVAs of the two experiments showed no significant effects involving experiment for S1 and S12, but for S2 there were interactions of experiment with A-pattern timing, *F*(2, 36) = 5.1, *p* = .019, and with B-pattern timing, *F*(2, 36) = 5.6, *p* = .010, due to more pronounced PCRs in Experiment 1 than in Experiment 2.

**Figure 5.**
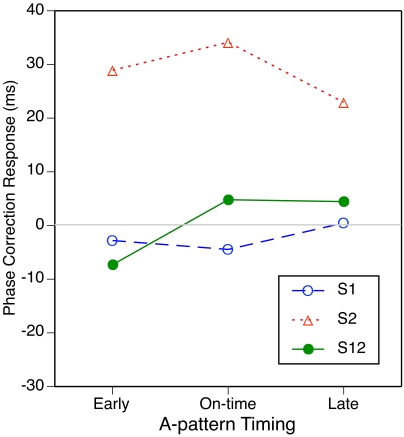
The mean phase correction response to the A-B transition when the B-pattern is empty, as a function of subdivision type and A-pattern timing.

Figure 5 shows the PCRs in the conditions that were new relative to Experiment 1 and in which an A-pattern of subdivisions was followed by empty IBIs (i.e., the A-pattern simply ended in the middle of the sequence). The PCRs are shown as a function of A-pattern timing. The results were striking and unexpected: Cessation of S2 elicited a large positive PCR regardless of S2 timing, whereas cessation of S1 or S12 elicited hardly any PCR at all. The main effect of subdivision type was highly significant, *F*(2, 18) = 41.7, *p* < .001, with no other effect approaching significance. We consider an interpretation in the General Discussion.

#### Pre-transition asynchronies and prediction of PCRs

**Figure 6.**
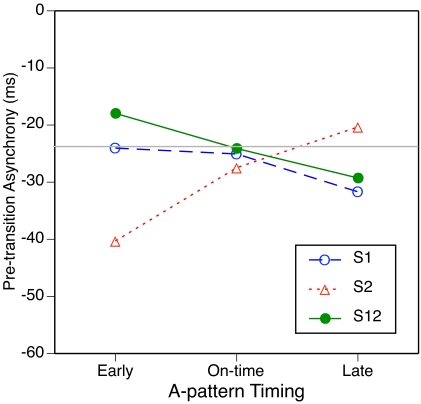
The mean pre-transition asynchrony as a function of subdivision type and A-pattern timing. The grey horizontal line represents the mean pre-probe asynchrony for the empty A-pattern.

Although we present a more detailed picture of asynchronies in later figures, we first show in Figure 6 the mean asynchrony of the tap immediately preceding the A-B transition, which can be compared directly with the pre-probe asynchrony in Experiment 1 (Figure 3). Here, differences among conditions were much less pronounced than they were in Experiment 1, again largely due to S1, which elicited more negative asynchronies here than in Experiment 1. As in Experiment 1, asynchronies were more negative for early than for late S2, whereas for S1 and S12 asynchronies tended to be less negative for early than for late timings. In the ANOVA, only the interaction was significant, *F*(4, 36) = 10.0, *p* < .001. In a joint ANOVA with Experiment 1, however, there were significant main effects of subdivision type, *F*(2, 36) = 9.9, *p* = .001, and of A-pattern (context), *F*(2, 36) = 4.5, *p* = .030, as well as a main effect of experiment, *F*(1, 18) = 4.5, *p* = .047, and an interaction of experiment with subdivision type, *F*(4, 36) = 9.9, *p* = .001.

As a final parallel to Experiment 1, the phase adaptation predicted from the pre-transition asynchronies and the actual change in timing across the A-B transition (expectancy violation) were used to predict the PCRs shown in Figure 4 (panels B-D). A stepwise multiple regression analysis yielded results very similar to those in Experiment 1: Expectancy violation accounted for 71% of the variance in the PCRs, whereas phase adaptation accounted for an additional 9%. According to the regression coefficients, the PCR could be described as constituting 15% of expectancy violation plus 42% of the (much smaller) phase adaptation.

It can also readily be seen that the PCRs in Figure 4 (A), which occur at the transition from an empty A-pattern to a B-pattern, cannot be predicted by considering the mean pre-transition asynchrony for an empty pattern in relation to the pre-transition asynchronies for various subdivision patterns (Figure 6). Moreover, the PCRs in Figure 5, which occur at the transition from an A-pattern to an empty B-pattern, can likewise not be predicted from the reverse relationship between the pre-transition asynchronies in Figure 6. In particular, the large positive PCRs to the cessation of an S2, regardless of timing, are not at all in line with the required phase adaptation suggested by the data in Figure 6.

#### Mean asynchronies

**Figure 7.**
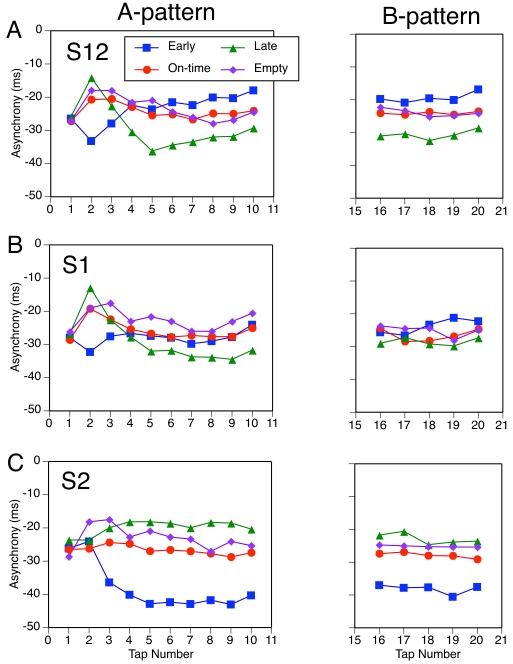
Mean asynchronies for A-patterns and the later part of B-patterns as a function of serial tap number.

In Figure 7, we present the mean asynchronies as a function of serial tap number, to show the temporal evolution of the tapping phase within each subdivision pattern. Taps 1-10 correspond to the A-pattern, and these asynchronies have been averaged here over the four B-patterns. Taps 16-20 correspond to the B-pattern, and these asynchronies have been averaged over the four A-patterns. Taps 11-15, which contain the PCR and subsequent phase adaptation to the B-pattern, have been excised here and are shown in the more detailed figures that are to follow.

Figure 7 enables us to make two points. First, the pre-transition asynchronies of tap 10 (Figure 6), which we used to predict phase adaptation, are representative of the effects of subdivision timing on the tapping phase both before and after the A-B transition. The mean asynchronies for A- and B-patterns generally join up well across the PCR gap (Taps 11-15), and there is little evidence of systematic phase drift. Second, it can be seen that it took about five taps to adapt the tapping phase to the A-patterns at the beginning of the sequence. Tap 1 had a similar mean asynchrony in all conditions because it preceded the first occurrence of the A-pattern. (Any carry-over effects from the preceding trial have been averaged out here.) The time course of adaptation to S12 and S1 (Figure 7 [A and B]) was very similar: Tap 2 exhibited an initial negative PCR to an early S12 or S1, a positive PCR to a late S12 or S1, and a positive but smaller PCR to an on-time S12 or S1 or to an empty IBI. Interestingly, although the PCRs to early and late subdivisions are consistent with the direction of the phase shift relative to the 1/3 and 2/3 points, they are contrary to the differences in asymptotic asynchronies from Tap 5 onward. Thus, the asynchrony trajectories cross over after Tap 3. Adaptation to S2 (Figure 7 [C]) was different: Tap 2 did not show any PCR, regardless of S2 timing, whereas Tap 3 showed an incipient change to the asymptotic asynchrony (which cannot be distinguished from a PCR in this case). This pattern of sequence-initial PCRs to A-patterns agrees well with the pattern of later PCRs to B-patterns following an empty A-pattern (Figure 4 [A]).

**Figure 8.**
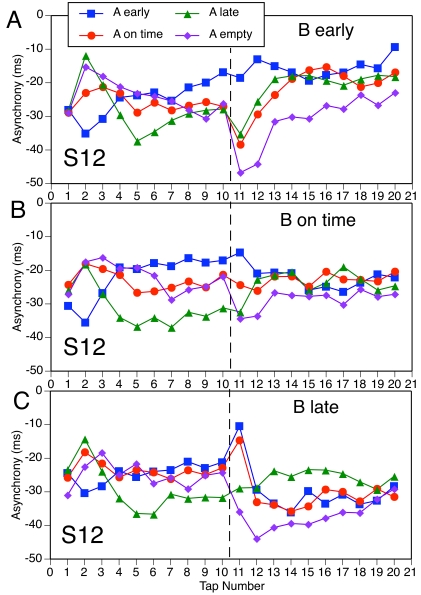
Mean asynchrony trajectories in all S12 conditions.

**Figure 9.**
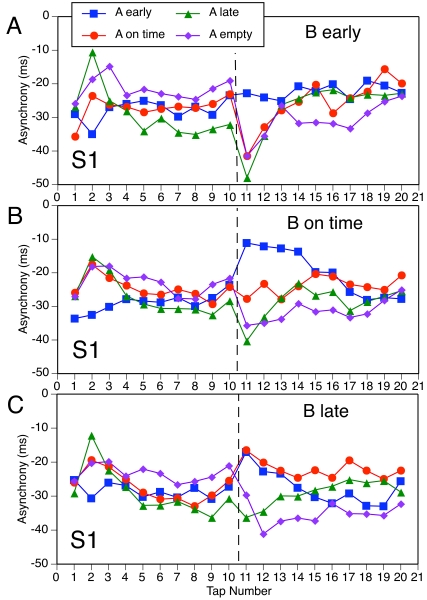
Mean asynchrony trajectories in all S1 conditions.

**Figure 10.**
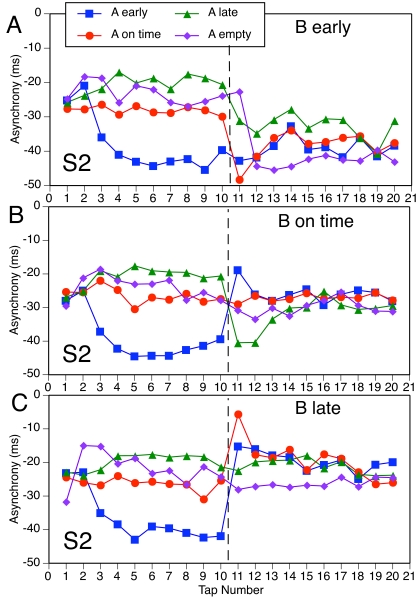
Mean asynchrony trajectories in all S2 conditions.

**Figure 11.**
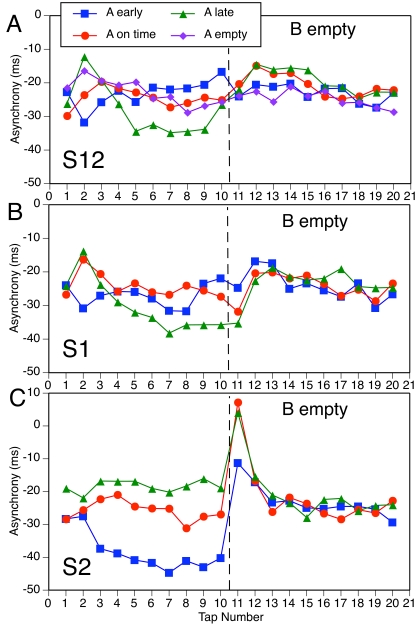
Mean asynchrony trajectories in all conditions with an empty B-pattern.

Finally, Figures 8-11 present the mean asynchronies of all taps in all conditions. There is greater variability here than in Figure 7 because each function is based on fewer data. The focus here is on the PCRs in relation to the A-B phase adaptation. As the previous regression analyses suggested, and as these figures make abundantly clear, the PCR is *not* part of the phase adaptation but rather is a nonlinearity superimposed on the trajectory of asynchronies. Only when the PCR and phase adaptation go in the same direction are they difficult to distinguish. Figures 8-11 also address the question of whether different A-patterns have any long-term effect on the asynchronies with B-patterns. For the sake of simplicity, we do not report statistical analyses of long-term effects (which would require separate tests at each sequence position) and restrict ourselves to qualitative observations.

Figure 8 shows all the S12 conditions, including the ones with an empty A-pattern. In three of the conditions the A- and B-patterns are the same, so there is neither a PCR nor phase adaptation. In four conditions (A on time, B early; A late, B early; A on time, B late; A early, B late), the PCR is clearly distinct from the phase adaptation, going in the opposite direction. In the conditions with empty A, there is a clear negative PCR that deviates from the rather minimal phase adaptation in two cases (B early, B on time) and seems to form part of a large phase adaptation in the third case (B late). The remaining two conditions (A early, B on time; A late, B on time) show a different pattern: There is no PCR, only a rapid phase adaptation at a delay of one tap (indistinguishable from a delayed PCR). Thus, it seems that an on-time B-pattern elicited a PCR only when the A-pattern was empty (cf. Figure 4), which suggests maintenance of on-time expectations for S12 in the face of a phase-shifted A-pattern. However, there is another way of interpreting these data. Suppose the phase adaptation was not delayed (and why should it be?) but started with Tap 11. A conservative estimate of the phase adaptation on Tap 11 could be obtained by interpolating between the asynchronies of taps 10 and 12; if phase adaptation were immediate, that would make the argument only stronger. Viewed against this predicted asynchrony, the actual asynchrony deviates in the direction the PCR would have been expected to go (i.e., positive for A early, B on time; negative for A late, B on time). Thus the apparent absence of a PCR can be understood as resulting from the cancellation of the PCR by simultaneous phase adaptation in the opposite direction. It need not be concluded, therefore, that participants’ expectations were not changed by shifted subdivisions in some conditions.

Some long-term effects of the A-pattern on adaptation to the B-pattern can be seen in Figure 8 (A and C): Compared to other A-patterns, an empty A-pattern made asynchronies with an early or late B-pattern more negative, and this effect lasted almost until the end of the sequence. There also appeared to be an extended effect of a late versus early or on-time A-pattern on asynchronies with a late B-pattern (Figure 8 [C]).

Turning to the S1 conditions in Figure 9, there are clear PCRs distinct from phase adaptation in all conditions except the three in which there was no phase shift and one (A empty, B late) in which the PCR can be seen as part of (i.e., goes in the same direction as) the phase adaptation. In some conditions (e.g., A late, B early), the PCR is contrary to the phase adaptation. There is also some evidence of long-lasting effects of the A-pattern, particularly of the empty pattern, on B-pattern asynchronies.

The S2 conditions in Figure 10 show a mixed pattern of results. In one condition (A empty, B early), there is no PCR but an abrupt phase adaptation after Tap 11. This cannot be interpreted as cancellation of a PCR by phase adaptation because they are expected to go in the same direction. In two other conditions (A late, B early; A early, B late), the PCR coincides with the phase adaptation. In two further conditions (A empty, B on time; A empty, B late), there is hardly any PCR but also hardly any phase adaptation. The absence of PCRs in the conditions with an empty A-pattern suggests that there was no latent expectation for S2. Only three conditions (A on time, B early; A early, B on time; A on time, B late) show a clear PCR that is distinct from the phase adaptation. Again, an empty A-pattern seemed to have long-term effects on B-pattern asynchronies when B was early or late.

Finally, consider the conditions in which an A-pattern changed to an empty B-pattern (Figure 11). For S12 and S1 (panels A and B), there were only very small PCRs, if any (cf. Figure 5). For S1, a phase adaptation followed Tap 11. For S2, by contrast, there were huge PCRs, even in a condition in which there was no phase adaptation to speak of (A on time, B empty). There were no indications of any long-term effects of the A-pattern here.

## General Discussion

Experiment 1 was motivated by two main questions: First, do (or can) musically trained listeners have latent expectations of triple subdivision of a beat? Second, can phase-shifted context shift the temporal expectations for triple subdivisions? Experiment 2 contributed additional data relevant to these questions but went beyond Experiment 1 in several ways, to be discussed later.

With regard to the first question, we acknowledge that we framed it within a restricted context. Because we found it unlikely that we would find evidence for latent expectations of triple subdivision in a context where duple subdivision could be expected, we deliberately embedded our “no-context” trials in a global experimental context in which triple subdivision was common. Thus, our question can be recast as “Do listeners have latent expectations of triple subdivision when the global experimental context encourages such expectations?” However, it also should be kept in mind that the triple subdivisions in other trials were often incomplete (S1 or S2) or temporally shifted (early or late). Thus the global context was certainly less expectancy inducing than a constant context of on-time triple (S12) subdivisions would have been.

With these qualifications, the results of both experiments suggest that participants did have (relatively weak) latent expectations for a subdivision at 1/3 of the IBI (S1, S12), but not for one at 2/3 of the IBI (S2). This conclusion is based on the fact that the PCR to the first occurrence of a subdivision depended on S1 timing (early, on-time, late) but not on S2 timing. Interpretation of these results is complicated by the fact that the PCRs were generally negative, which seemed to be a nonspecific reaction to the probe (Experiment 1) or B-pattern onset (Experiment 2). The relative weakness of the latent expectations for S1can be attributed to the variability of the global context. However, the absence of any latent temporal expectations for S2 is surprising, not only because PCRs were larger for S2 than for S1 in the context conditions of Experiment 1 but also because in real music S2 frequently occurs by itself, whereas S1 rarely does. It may be the case, however, that S1, when it does occur by itself, is usually timed precisely, whereas S2 is often subject to large deviations from precise timing (London, 2004, pp. 37, 171). In the Introduction, we mentioned the swing rhythm of jazz as an example. If musical experience leads to a mental representation of the distribution of rhythmic interval ratios encountered in the past (Sadakata, Desain, & Honing, 2006), then latent expectations for S2 may well be poorly defined, whereas those for S1 may be weak but precise. It could be that such general musical experience is reflected in the results of our no-context (or empty A-pattern) condition.

The answer to the second question, whether phase-shifted context would shift participants’ temporal expectations for triple subdivisions, is clearly positive. In Experiment 1, following merely five repetitions of phase-shifted subdivisions, participants clearly expected subdivisions to continue with the same timings, with the possible exception of late S12 context (but see below). These expectations were reflected in PCRs that depended on the direction and magnitude of the phase shift. If participants had instead maintained fixed expectations for subdivisions to occur at the 1/3 and 2/3 points, their PCRs to the probe should have been either unaffected by preceding context (which clearly was not the case) or explained fully as an incipient change from the context-specific mean asynchrony to the probe-specific mean asynchrony. Although the predicted phase adaptation made a significant contribution to the manifest PCR, the magnitude of the temporal shift between context and probe, which quantified temporal expectancy violation, was a much stronger predictor. This result suggests that the PCR is indeed a response to cognitive expectancy violation and is largely separate from the contingencies of phase adaptation that presumably arise on the level of rhythmic motor entrainment.

Experiment 2 confirmed these findings, although there were some unexpected differences in results. Even though the context (A-pattern) was more extensive in Experiment 2 (nine repetitions), PCRs tended to be smaller than in Experiment 1, especially for S2. The reason for this difference is unclear. If anything, PCRs might have been expected to be smaller in Experiment 1 because adaptation to the context may have been still incomplete when the probe occurred. The asynchrony trajectories for the A-patterns in Experiment 2 suggest, however, that adaptation was complete after about five taps, and therefore should also have been complete in Experiment 1 when the probe occurred. In Experiment 2 it also seemed that expectations for S12 did not adapt to phase-shifted A-patterns, although expectations for S1 and S2 did. This impression, however, seemed to be the result of PCRs and phase adaptation tending in opposite directions, so that cancellation occurred. On the whole, the agreement between experiments was more striking than were the differences.

We consider our most important result the demonstration that the PCR in the present paradigm depends much more on the magnitude of the physical phase shift between context and probe (the expectancy violation) than on the phase shift required in the taps in order to adapt to a new context (the probe or B-pattern). The asynchrony trajectories obtained in Experiment 2 reveal that, in most cases, the PCR is a pronounced local nonlinearity in the phase adaptation, indeed a superimposed effect of independent origin. Only in some conditions was the PCR indistinguishable from the phase adaptation, usually when they had the same direction. It is important to emphasize that the PCR studied here is different from the PCR investigated in most previous studies (reviewed in Repp, 2005). Usually, participants synchronize their taps with a beat that is perturbed, and the PCR is the reaction to that perturbation. Here, however, participants synchronized with a fixed beat, and the intervening subdivisions were perturbed. In the traditional paradigm, the PCR is assumed to be the beginning of the phase adaptation of the taps: If the phase of the beat is shifted, the phase of the taps must follow suit in order to re-establish synchrony (typically with the same mean asynchrony). There is no evidence in those earlier studies that the PCR is separate from the phase adaptation, which usually follows the exponential shape predicted by a linear model of phase correction (Vorberg & Schulze, 2002). In the present paradigm, by contrast, the tapping phase (mean asynchrony) is affected by a phase shift of subdivisions, which necessitates a phase adaptation in the taps. However, as we have shown, the PCR elicited by the phase perturbation is generally *not* the initial part of this phase adaptation and often goes in the opposite direction. It emerges from the present results as a separate, largely independent reaction to the physical phase shift. We attribute this reaction to the violation of temporal expectancies induced by the preceding subdivision pattern (context or A-pattern). Basically, unexpectedly early or late subdivisions led to an automatic expectation that the beat (the synchronization target) will also occur early or late, and the PCR is triggered by that expectation. The phase adaptation, by contrast, does not depend on expectations but only on the phase relation between fixed beats and subdivisions.

Expectancy violation accounts best for the PCR to moderate phase shifts (60 ms in our experiments, or 1/12 of the IBI). The PCRs to larger phase shifts (120 ms, or 1/6 of the IBI) tended to be smaller than the increased size of the phase shift would lead one to expect. This may have occurred because the PCR increases nonlinearly with perturbation magnitude (cf. Repp, 2002a, 2002b) or possibly because one of the subdivisions (early S1 or late S2) coincided with a quadruple subdivision point (1/4 of the IBI) and therefore seemed less deviant. Expectancy violation cannot account easily, however, for one striking result of Experiment 2: the large positive PCR to the cessation of a S2 pattern, regardless of its timing. That response may have been due to *perceptual grouping* of S2 with the following beat. Participants may have been entrained to make their taps at a certain time after the S2 onset. If S2 was suddenly missing, they may have timed their next tap from the moment the absence of S2 became evident, resulting in a positive PCR (delayed tap). The fact that the cessation of S12 did not cause a large PCR suggests that S2 was not grouped with the following beat when S1 was also present. Perceptual grouping could conceivably also explain the apparent absence of latent expectations for S2, although it is not quite clear how grouping would efface the PCR.

The analysis of pre-probe asynchronies in Experiment 1 and the more extensive analyses of asynchrony trajectories in Experiment 2 reveal that the timing of subdivisions has systematic effects on the tapping phase (mean asynchronies) in synchronization with a fixed beat. How should these effects be explained? One possibility is that they represent an attraction of the taps to the nearest subdivision tone. Attraction of taps to distractor tones, especially leading tones, has been demonstrated in previous studies (Hove, Keller, & Krumhansl, 2007; Repp, 2003b, 2004), but it tended to occur only when the target and distractor tones were within about 150 ms of each other. In the present study, subdivision tones came only as close as 180 ms to the beat (early S1 or late S2), which should have lead to little or no attraction. Moreover, an early S1 should have led to positive (or less negative) asynchronies, whereas a late S2 should have caused larger negative asynchronies. A glance at Figure 6 or Figure 7 reveals that both predictions are incorrect: S2 timing exerted the largest effects on asynchronies, with the most negative values for early S2 and the least negative values for late S2. S12 timing had a less pronounced effect in the opposite direction. S1 timing had the smallest effects, similar to those of S12, at least in Experiment 2. These effects are best understood as (small and involuntary) sensorimotor adjustments to distortions of the expected interval ratios for triple subdivision. The opposite shifts for S2 compared to S1 and S12 again suggest that S2 was perceptually grouped with the following beat when it occurred by itself, but not when it occurred together with S1. A more thorough exploration and explanation of these effects may require coupled-oscillator models that take into account the multiple resonance frequencies induced by a non-isochronous rhythm (see Tomic & Janata, 2008).

In Experiment 2, on-time subdivisions had little effect on asynchronies compared to empty IBIs, which may be taken as an indication that this timing of the subdivisions was perceived as natural. In Experiment 1, there were some differences between these two conditions that, however, are difficult to interpret. It is possible that exact isochrony is not perceptually optimal in the case of triple subdivision.

One effect that was *not* observed in the present experiments is a general reduction of negative asynchronies when any subdivisions occurred between beats. Such a reduction is predicted by the hypothesis (Wohlschläger & Koch, 2000) that empty IBIs are generally underestimated, which causes negative asynchronies. Repp (2008b) reports related findings that likewise do not support the perceptual underestimation hypothesis.

One final comment is in order. In this paper we have considered the PCR as a response to expectancy violation, which seems to imply that a phase-shifted subdivision tone is compared to its expected temporal position, and if a discrepancy is detected, a PCR is triggered. One of us, however, has long argued against the hypothesis that the PCR is triggered by perception of asynchronies (see, e.g., Repp, 2005), and the discrepancy between an expectation and an actual tone onset is a kind of asynchrony. Rather, he has argued that taps are timed with reference to recent tones, with the timed interval arising from an internal model (memory representation) of the pacing rhythm. Thus, no actual comparison of expected and observed onset times is needed; it is sufficient to assume phase resetting of taps with reference to preceding tone(s). An internal model of a rhythm implies expectations, however, and thus is compatible with a discussion in terms of expectations, as long as it is understood that expectancy violation does not have to be consciously perceived in order for a PCR to occur.

In summary, the present results suggest that, far from being tied to simple interval ratios, temporal expectations for subdivisions of a beat are flexible and context-sensitive. Basically, listeners quickly come to expect whatever rhythm they hear repeatedly and react automatically to deviations from these expectations, even if the deviation represents a return to isochronous timing. Participants’ sensitivity to deviations from arbitrary interval ratios, observed here in a study of perceptually guided action, contrasts with the often demonstrated difficulties even musically trained participants have with perceptually judging or (re)producing complex interval ratios (Collier & Wright, 1995; Povel, 1981; Semjen & Ivry, 2001; Sternberg, Knoll, & Zukofsky, 1982). Although direct comparisons remain to be conducted, perhaps we have found here another dissociation between conscious perception of timing and the on-line perceptual guidance of action (Repp, 2000, 2006, 2009).
